# Tanshinone IIA attenuates ovalbumin-induced airway inflammation and hyperresponsiveness in a murine model of asthma

**DOI:** 10.22038/ijbms.2018.30598.7375

**Published:** 2019-02

**Authors:** Shi-Biao Wang, Xiao-Feng Guo, Bin Weng, Su-Ping Tang, Hui-Jie Zhang

**Affiliations:** 1 Department of Pediatric, Fujian Provincial Meternity and Children’s Hospital of Fujian Medical University, Fuzhou, Fujian 350001, China; 2 Department of Allergy, Fuzhou Children’s Hospital, Teaching Hospital of Fujian Medical University, Fuzhou, Fujian 350005, China

**Keywords:** Asthma, Inflammation, Nuclear factor-κB, Oxidative stress, Tanshinone IIA

## Abstract

**Objective(s):**

Tanshinone IIA (T. IIA), one of the most pharmacologically active components extracted from *Salviae miltiorrhiza*, has anti-inflammatory and antioxidant features. The aim of the present study is to investigate the benefit of *T. IIA* on asthma using a murine model of asthma induced by ovalbumin (OVA).

**Materials and Methods:**

Male BALB/c mice were used in the present study. The mice were sensitized by OVA intraperitoneal injection on days 0 and 14, and received aerosolized OVA challenge for 30 min daily on days 21-23. T. IIA (10 mg/kg twice daily) intraperitoneal injection was performed on days 18-23.

**Results:**

Treatment of T. IIA reduced the levels of interleukin (IL)-4, IL-5, and IL-13 in bronchoalveolar lavage fluid (BALF) (*P*<0.05 for all cases). The OVA-induced elevation of total white blood cells as well as differential white blood cells in BALF and blood were inhibited by* T. IIA* (*P*<0.05 for all cases). Moreover, airway hyperresponsiveness was dampened in T. IIA-treated group (*P*<0.05). T. IIA inhibited the activation of nuclear factor-κB in asthmatic mice (*P*<0.05). The activity of nuclear factor erythroid-2-related factor 2 was enhanced in T. IIA-treated group (*P*<0.05). T. IIA elevated the activities of heme oxygenase-1, glutathione peroxidase, and superoxide dismutase (*P*<0.05 for all cases).

**Conclusion:**

T. IIA inhibits OVA-induced airway inflammation and hyperresponsiveness. T. IIA is a potential therapeutic agent for asthma.

## Introduction

Airway hyper-responsiveness and inflammation are the most common features of asthma (1). Cytokines from T helper 2 (Th2) cells is believed to play a vital effect in organizing the chronic inflammation of asthma (1). Inhibition of inflammation is an important strategy for pulmonary inflammatory disorders (2-4). 

Nuclear factor erythroid-2-related factor 2 (Nrf2), a transcription factor, is known as a vital antioxidant defense mechanism. Scientific evidence has shown that Nrf2 is critical in protecting the lung against oxidative stress in asthma (5). Nuclear factor-κB (NF-κB), another important transcription factor, is believed to play a vital effect in organizing the expression of cytokines in pulmonary diseases (4). Inhibition of NF-κB has shown beneficial effect on asthma (6). Inhibition of NF-κB and activation of Nrf2 are associated with dampened airway inflammation and hyperresponsiveness in asthma (4, 5). Thus, induction of Nrf2 and inhibition of NF-κB are potential strategy for reduction of asthma.

Despite significant advances in the management of asthma, novel treatments for asthma are still required as the current strategies have their limitations (7-9). Recently, there are a growing interest on herbal medicines and natural products (10, 11). Tanshinone IIA (T.IIA) is a pharmacologically active component of *Salviae miltiorrhizae, *which is a traditional Chinese medicine and has antioxidant and anti-inflammation features (12-15). Oxidative stress-induced myocardial apoptosis was inhibited by T. IIA (16). T. IIA dampened lipopolysaccharide-induced pulmonary inflammation and edema in an animal model of acute lung injury (17). T. IIA is believed to play a beneficial role on chronic obstructive pulmonary disease (18). However, the effect of T. IIA on asthma remains unclear. The current study investigates the effect of T. IIA on ovalbumin (OVA)-induced airway inflammation and hyperresponsiveness using a murine model of asthma. 

## Materials and Methods

***Animals***

All experiments were conducted in accordance with the Helsinki convention for the use and care of animals. All experiment protocols were reviewed and approved by the Research Ethics Committee of Fujian Medical University.

Six-week old male BALB/c mice (obtained from the Experimental Animal Center of Fujian Medical University) were bred in a specific pathogen-free and temperature controlled (22±2 ^°^C) animal facility. The mice were maintained on a 12 hr light/ 12 hr dark schedule and received standard laboratory rodent chow and tap drinking water *ad libitum*. 

***Sensitization and provocation protocols***

The sensitization and provocation protocols used in the present study were discussed previously (19). Mice were immunized using OVA (sigma-Aldrich, St Louis, MO, USA) intraperitoneal injection on days 0 and 14. OVA (50 μl) and an adjuvant, Al(OH)_3_ (50 μl, Pierce, Rockford, IL, USA), were dissolved in normal saline (NS; 200 μl) before use. From the day 21 to 23, animals were exposed to aerosolized OVA (1% OVA) for 30 min once daily. Mice in control group received the equivalent Al(OH)_3_ diluted in NS intraperitoneal injection, and exposed to a nebulized aerosol of NS at the same time points as the OVA challenged animals.

***T. IIA administration***

T. IIA was obtained from Shanghai No. 1 Biochemical Pharmaceutical Co, Ltd. (Shanghai, China). The animals were randomly divided into various groups: control (sham+ NS), T. IIA control (sham+T. IIA), asthma+NS, and asthma+T. IIA group. Briefly, the mice were treated with intraperitoneal injection of 10 mg/kg T. IIA twice daily on days 18-23. The dose of T. IIA selected in the present study was based on previously published articles (13, 16) and our preliminary study (data not shown). Equivalent NS was administrated for control group. 

***Bronchoalveolar lavage fluid (BALF) analysis***

The mice were anesthetized (50 mg/kg thiopental intraperitoneal injection), and the trachea was cannulated with a 0.6 mm catheter and secured with a silk suture. Sterile NS (1 ml) was instilled through the catheter using a 1 mL syringe for 3 times. More than 90% of BALF was withdrawn. Then, BALF centrifugation was performed (1,200 rpm for 5 min at 4 ^°^C) using a cytocentrifugation (cytospin 3, Shandon Instruments, Pittsburgh, PA). The sediment cells were washed and stained with Giemsa stain. Total and different subtypes of white blood cells were counted with a hemocytometer. The supernatant was analyzed for cytokines.

***Proinflammatory cytokines measurement***

Commercial enzyme-linked immunosorbent assay (ELISA) kits were used to measure the proinflammatory cytokine levels, including interleukin (IL)-4, IL-5, as well as IL-13, in BALF following the manufacturer’s protocol (eBioscience Co, San Diego, USA.). 

***Airway responsiveness measurement***

The mice were anesthetized (50 mg/kg thiopental intraperitoneal injection) and mechanically ventilated with a rodent ventilator at 24 hr after the last aerosolized OVA or vehicle challenge. The mice were challenged with aerosolized methacholine (12.5, 25, 50 mg/ml; Sigma-Aldrich) or NS after being stabilized (19). Then, tissue resistance, tissue elastance, respiratory system elastance, respiratory system resistance, and airway resistance were measured (Buxco Research System). 

***NF-κB and Nrf2 activity analysis***

A nuclear extract kit (Active Motif North America) was used to prepare nuclear extracts from lung tissues. NF-κB p65 binding activity was detected by using an ELISA assay kit (Active Motif, Carlsbad, CA, USA) following the manufacturer’s protocol (20). Briefly, the nuclear extracts were incubated with the p65 subunit of NF-κB consensus site oligonucleotides (5`-GGGACTTTCC-3`) immobilized to 96-well plates. The DNA binding activity of NF-κB p65 was detected with an antibody specific to the activated form of NF-κB p65 and visualized by colorimetric reaction catalyzed by horseradish peroxidase-conjugated secondary antibody, and absorbance was measured at 450 nm with a reference wavelength of 655 nm.

A TransAM Nrf2 assay kit (Active Motif, Carlsbad, CA, USA) was used to measure the Nrf2 binding activity as described previously (21). Briefly, the nuclear extracts were incubated in 96-well plates coated with immobilized oligonucleotide containing a consensus (5`-GTCACAGTGACTCAGCAGAATCTG-3`) binding site for antioxidant response element. The Nrf2 binding activity to the target oligonucleotide was detected by incubation with primary antibody specific for DNA-bound Nrf2, visualized by horseradish peroxidase conjugate and developing solution, and quantified at 405 nm. 

***Heme oxygenase (HO)-1 activity assay***

The HO-1 activity in tissue homogenate was determined by measuring the generated bilirubin as previously described (22). Briefly, samples of lung tissue were added to a mixture containing glucose 6-phosphate, glucose 6-phosphate dehydrogenase, protohemin, and nicotinamide adenine dinucleotide phosphate. The reaction was continued for 1 hr at 37˚C. The bilirubin level was determined by a detection reader at excitation and emission wavelengths of 464 and 530 nm, respectively.

***Antioxidant enzymes activities assay***

A glutathione peroxidase (GPx) activity detection kit (Nanjing Jiancheng Bioengineering Institute, Jiangsu, China) was used to measure the GPx activity in lung homogenate. The GPx activity was determined by measuring the level of oxidized glutathione (GSSG), which was converted from glutathione (GSH) by GPx. The sample of lung tissue was incubated with H_2_O_2_. The absorbance of GSSG was measured at 412 nm. 

A superoxide dismutase (SOD) activity detection kit (Nanjing Jiancheng Bioengineering Institute, Jiangsu, China) was used to measure the SOD activity in lung homogenate. The level of formazan salt was used as an indicator of SOD activity. Briefly, sample of lung tissue was added to a mixture including tetrazolium salt and xanthine oxidase enzyme at 37 ^°^C. The reaction was continued for 20 min. The absorbance of formazan salt was detected at 550 nm. 

***Maleic dialdehyde (MDA) production measurement***

The MDA production was used to indicate reactive oxygen species (ROS) levels in pulmonary tissues. The sample of lung tissue was added to a mixture containing acetic acid, sodium dodecyl sulfate, aqueous solution of thio-barbituric acid, n-butanol, and pyridine. The mixture was shaken and centrifuged (4,000 rpm for 10 min). The MDA production was determined at 532 nm by a detection reader.

***White blood cell analysis***

Blood sample was collected by cardiac puncture under anesthesia (50 mg/kg ketamine intraperitoneal injection) with a heparinized syringe (5 ml) and analyzed as described previously (23).

***Statistical analysis***

All data were presented as mean±SEM. Differences in values were measured by one-way analysis of variance (ANOVA) followed by the Student-Newman-Keuls method, and were considered statistically significant if* P *value less than 0.05. SPSS 19.0 software (IBM, Armonk, USA) was used for all statistical analysis.

## Results

***T. IIA reduces airway inflammation***

**Figure 1:**
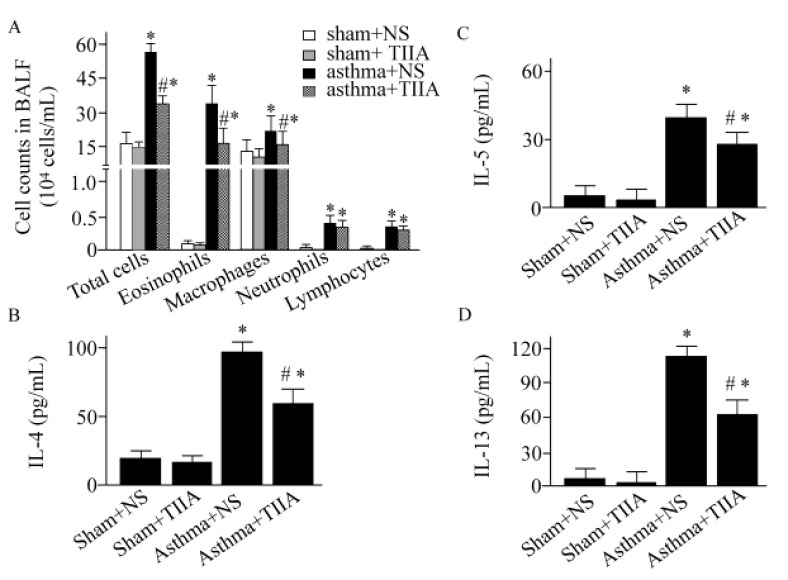
Tanshinone IIA reduces inflammatory cells and proinflammatory cytokines in bronchoalveolar lavage fluid. Values are expressed as mean±SEM. * *P*<0.05, compared to Sham+ normal saline (NS) group; #*P*<0.05, compared to asthma+NS group. TIIA, Tanshinone IIA; BALF, bronchoalveolar lavage fluid; IL-4, interleukin -4; IL-5, interleukin -5; IL-13, interleukin-13

The total and differential white blood cells counts were shown in Figure 1A and Table 1. As indicated in Figure 1A, compared with control animals, the number of total inflammatory cells in BALF was increased in asthmatic mice (*P*<0.05). Meanwhile, the number of eosinophils in BALF was elevated in asthmatic mice (*P *< 0.05). In blood, the number of total inflammatory cells was increased by 1.74-fold in vehicle-treated asthmatic mice compared to control (*P*<0.05), (Table 1). Similarly, the number of eosinophils in blood was elevated by 2.4-fold (*P*<0.05), (Table 1). T. IIA inhibited the total inflammatory cells and eosinophils in blood and infiltration in BALF (*P*<0.05 for all cases), (Table 1; Figure 1A). The IL-4 level in BALF was increased by 6-fold in asthmatic mice (*P*<0.05), (Figure 1B). The OVA-induced elevating of IL-4 was inhibited by T. IIA treatment (*P*<0.05), (Figure 1B). Moreover, T. IIA treatment decreased IL-5 and IL-13 levels by 38% and 45%, respectively (*P*<0.05 for both cases), (Figure 1C and D).

**Figure 2:**
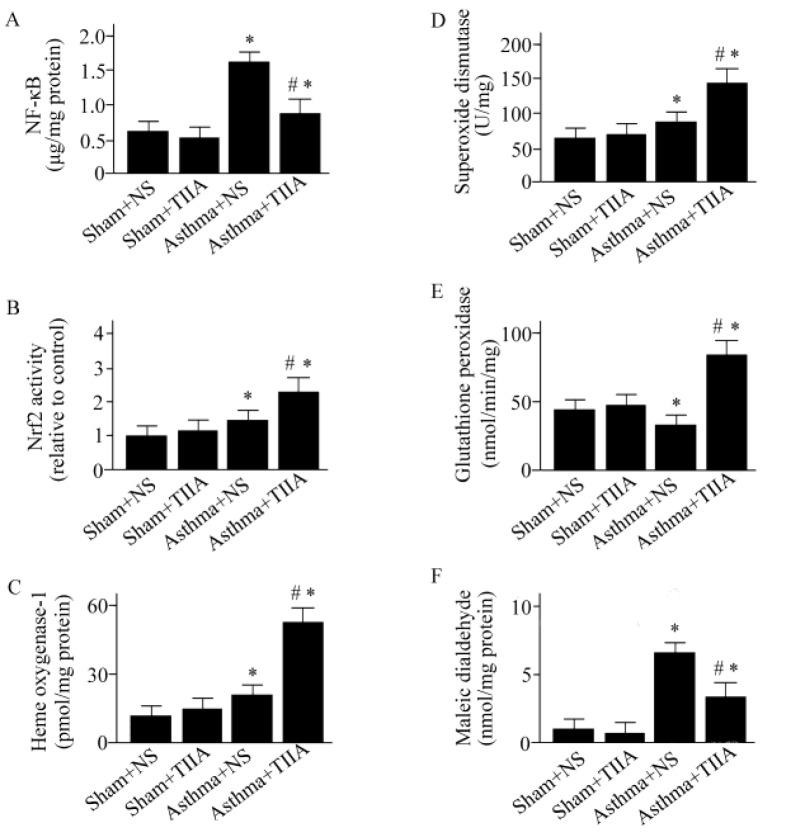
Effects of Tanshinone IIA on respiratory system elastance (A), respiratory system resistance (B), airway resistance (C), tissue elastance (D), and tissue resistance (E). Values are expressed as mean±SEM. * *P*<0.05, compared to Sham+ normal saline (NS) group; #*P*<0.05, compared to asthma+NS group. T. IIA, Tanshinone IIA; Ers, respiratory system elastance; Rrs, respiratory system resistance

**Figure 3:**
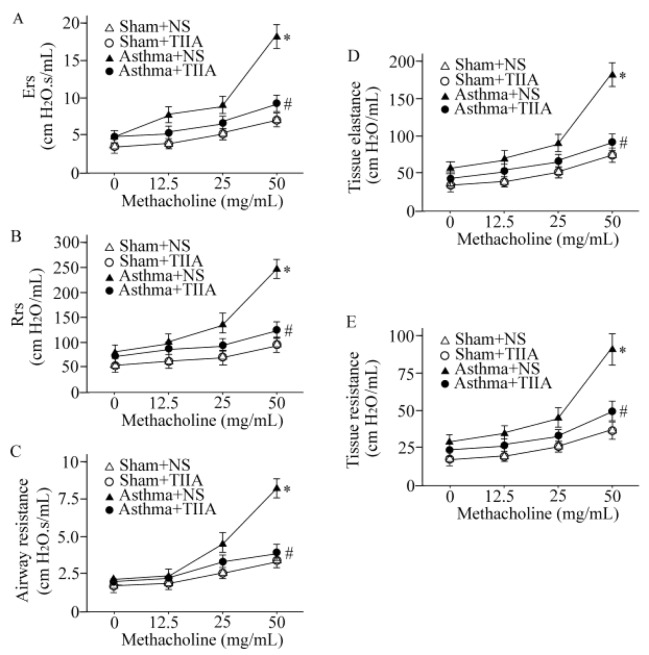
Effects of Tanshinone IIA on nuclear factor-κB activity (A), nuclear factor erythroid-2-related factor 2 activity (B), heme oxygenase-1 activity (C), superoxide dismutase activity (D), glutathione peroxidase activity (E), and maleic dialdehyde production (F). Values are shown as mean±SEM. *P<0.05, compared to Sham+normal saline (NS) group; # *P*<0.05, compared to asthma+NS group. T. IIA*, *Tanshinone IIA; NF-κB, nuclear factor-κB; Nrf2, nuclear factor erythroid-2-related factor 2

**Table 1:** Effect of *Tanshinone IIA* on total blood and differential white blood cells counts (×10^3^ cells/ml)

	sham+NS	sham+*T. IIA*	asthma+NS	asthma+*T. IIA*
Total cells	6.23±1.57	6.01±1.35	10.81±2.13*	7.83±1.53#
Lymphocytes	4.23±0.78	4.02±0.91	6.26±1.46*	4.96±0.74#
Neutrophils	1.55±0.17	1.56±0.22	3.45±0.66*	2.23±0.35#
Eosinophils	0.21±0.09	0.18±0.10	0.50±0.23*	0.29±0.18#
Monocytes	0.20±0.12	0.21±0.07	0.46±0.37*	0.32±0.15#

Values are as mean±SEM.

* *P* < 0.05, compared to Sham+normal saline (NS) group;

# *P* < 0.05, compared to asthma+NS group. *T. IIA*, *Tanshinone IIA*.

***Effect of T. IIA on airway hyperresponsiveness***

We performed a methacholine dose–response curve to evaluate if T. IIA protects lung against airway hyperresponsiveness. As shown in Figure 2, tissue resistance, tissue elastance, respiratory system elastance, respiratory system resistance, and airway resistance were increased in OVA-sensitized mice challenged with methacholine (*P*<0.05 for all cases). Our results showed that T. IIA markedly inhibited airway hyperresponsiveness compared to vehicle-treated asthmatic mice (*P*<0.05 for all cases), (Figure 2A, B, C, D, and E).

***T. IIA inhibits NF-κB activation and elevates Nrf2 activity***

The activity of NF-κB was increased in OVA-treated mice (*P*<0.05), (Figure 3A). T. IIA treatment decreased the activity of NF-κB by 52% compared to the vehicle-treated asthmatic mice (*P*<0.05), (Figure 3A). Moreover, T. IIA enhanced Nrf2 activity in asthmatic mice compared to vehicle-treated animals (*P*<0.05), (Figure 3B).

***Effect of T. IIA on antioxidant enzymes activities and ROS production***

The activities of GPx, SOD, and HO-1 were enhanced in T. IIA*-*treated asthmatic animals (*P*<0.05 for all cases), (Figure 3C, D, and E). Moreover, T. IIA treatment inhibited the OVA-induced ROS generation compared to vehicle-treated asthmatic animals (*P*<0.05), (Figure 3F).

## Discussion

Inflammation is believed to play a vital role in asthma (1). Thus, inhibition of inflammation is believed to be a fundamental strategy for controlling asthma. In the present study, T. IIA inhibited the infiltration of inflammatory cells in the lung, reduced the productions of IL-4, IL-5, and IL-13, and dampened airway hyperresponsiveness. Moreover, T. IIA inhibited NF-κB activation, and elevated the activities of Nrf2 and antioxidant enzymes. The ROS production was reduced in T. IIA-treated group. These results suggest that T. IIA has benefit on OVA-induced asthma.

Activation of NF-κB appears to play a vital effect in the pathogenesis of pulmonary inflammatory disorders (4, 24). Accumulating evidences have indicated that inhibition of NF-κB has a benefit on asthma (4, 6). Evidences have shown that T. IIA inhibits the activation of NF-κB (25-28). T. IIA attenuates ischemia/reperfusion injury caused by liver grafts via down-regulation of the NF-κB pathway (29). Phosphorylated NF-κB and IκBα in abdominal aortic aneurysm induced by elastase perfusion were decreased by T. IIA treatment (30). The present results showed that T. IIA dampened NF-κB activation in asthmatic mice. This result suggests that inhibition of NF-κB is involved in the protective effect of T. IIA on asthma.

IL-4, IL-5, and IL-13 belong to Th2 cytokines, which play a fundamental effect in asthma (31-33). Evidences have shown that IL-4 exacerbated asthma via induction of autophagy in B cells (34). Deletion of IL-4 or IL-13 using monoclonal antibodies has shown a benefit on asthma control (35). B cells and eosinophils exert a vital effect in asthma. Evidences have shown that IL-5 exerts an important role on maturation and differentiation of B cells and eosinophils (36). Our results showed that the levels of IL-4, IL-5, as well as IL-13 were reduced in T. IIA*-*treated asthmatic mice. Our findings, combined with previous data, suggest that the benefits of T. IIA on asthma are associated with its effect on inhibition of Th2 cytokines.

Oxidative stress is believed to play a notable role in the pathogenesis of asthma (37, 38). Inhibition of oxidative stress is associated with dampened asthma (37, 38). Nrf2 is a major transcription factor that regulates the expression of antioxidants (39). GPx and SOD are important antioxidants against asthma (40-43). SOD and GPx activities were elevated in T. IIA*-*treated rats with liver steatosis (44). Our results showed that the activities of SOD and GPx were up-regulated by T. IIA in asthmatic mice. 

Aside from the GPx and SOD, T. IIA also induced an increase in HO-1 activity in the present study. HO-1 is known as a cytoprotective enzyme (45). HO-1 plays an important role in maintaining cellular homeostasis (45). HO-1 reduces airway inflammation induced by OVA via inhibition of immune response that is mediated by Th17 cell (46, 47). Consistent with our study, evidences have shown that induction of HO-1 reduces airway inflammation induced by OVA (48).

MDA is a commonly used indicator of oxidative stress (49). Increased MDA levels were found in adult and children patients with asthma (50, 51). Our results showed that the MDA levels were elevated in asthmatic mice. Moreover, our results showed that the OVA-induced elevation of MDA was inhibited in T. IIA*-*treated asthmatic mice. This result suggests a benefit of T. IIA in regulating the equilibrium of oxidant-antioxidant in asthma.

Our findings, combined with previous data, suggest that T. IIA has antioxidant effect (16, 44). Nrf2 plays a vital effect on reduction of oxidative stress (39). Our results showed that the Nrf2 activity is increased in asthmatic mice. The elevated Nrf2 activity is a stress response of the body defense system. T. IIA treatment resulted in a further elevation of Nrf2 activity. Nrf2-regulated genes have a low basal expression, as Nrf2 binds to Kelch-like ECH-associated protein 1 (Keap1), an inhibitor of Nrf2, normally. When released from Keap1, Nrf2 binds to target genes in the nucleus (52). Findings have shown that T. IIA induced the degradation of Keap1, and up-regulated Nrf2 gene transcription (53). Our results suggest that the up-regulation of Nrf2 and antioxidant enzyme activities are involved in the antioxidant effect of T. IIA on asthmatic mice. 

## Conclusion

The current results suggest that T. IIA inhibits OVA-induced airway inflammation and hyperresponsiveness. T. IIA is a potential therapeutic agent for asthma.
